# A Review on the Incorporation of Diatomaceous Earth as a Geopolymer-Based Concrete Building Resource

**DOI:** 10.3390/ma15207130

**Published:** 2022-10-13

**Authors:** Janet J. Kipsanai, Paul M. Wambua, Saul S. Namango, Sofiane Amziane

**Affiliations:** 1 Department of Mechanical and Production Engineering, School of Engineering, Moi University, Eldoret 30100, Kenya; 2 Professor of Materials Engineering, Department of Manufacturing, Industrial and Textile Engineering, School of Engineering, Moi University, Eldoret 30100, Kenya; 3 Associate Professor of Chemical & Processing Engineering, Department of Chemical and Process Engineering, School of Engineering, Moi University, Eldoret 30100, Kenya; 4 Institut Pascal, Université Clermont Auvergne, CNRS, INP, Clermont, F-63000 Clermont-Ferrand, France

**Keywords:** building material, sustainability, geopolymers, diatomaceous earth, performance properties

## Abstract

The development of geopolymer building composites at a lower cost with a smaller carbon footprint may lessen the growing concerns about global warming brought on by emissions of a critical greenhouse gas (CO_2_) paired with the high production costs in the cement sector. Diatomaceous earth, commonly used as an admixture or partial replacement of cement owing to its most effective pozzolanic properties, has been investigated as a precursor in geopolymer concrete development. Several studies have been examined to develop a greater understanding of its characterization, inclusion status, and impacts on the performance aspects of concrete. The literature review showed that using diatomaceous earth is one of the effective ways to create sustainable, insulating, lightweight building materials while minimizing the harmful economic and environmental effects of industrial solid wastes. However, since most studies have focused on its integration as a partial cement substitute or a replacement for fine aggregate, further research on diatomaceous earth-based clinker-free concrete is required. A lack of research on geopolymer concrete’s reinforcement with either natural or synthetic fibers, or a combination of the two, was also discovered. This review also showed that there has been remarkably little effort made towards theoretical property correlation modeling for predicting concrete performance. It is anticipated that the detailed overview presented herein will guide potential researchers in defining their future paths in the study area.

## 1. Introduction

The construction sector is essential to sustainable development since it contributes significantly to a country’s economy and its activities are critical to achieving the socio-economic development goals of providing housing, infrastructure, and employment [1,2,3]. Building and construction have been found to consume more than 40% of the world’s energy while emitting roughly the same amount of CO_2_ [4,5,6] as a result of the widespread use of cement-based concrete [7,8], which has led to an estimated global production of around 25 billion tonnes per year [9,10,11]. This eco-footprint is also expected to rise with the significant population growth anticipated by 2050 [3]. Currently, CO_2_ emissions are to blame for 65% of global warming, with ordinary Portland cement (OPC) production contributing around 8% of all CO_2_-related greenhouse gas emissions [12,13]. The negative impacts of using cement-based concrete and ceramic bricks can be mitigated by adopting sustainable practices.

**Figure 1:**
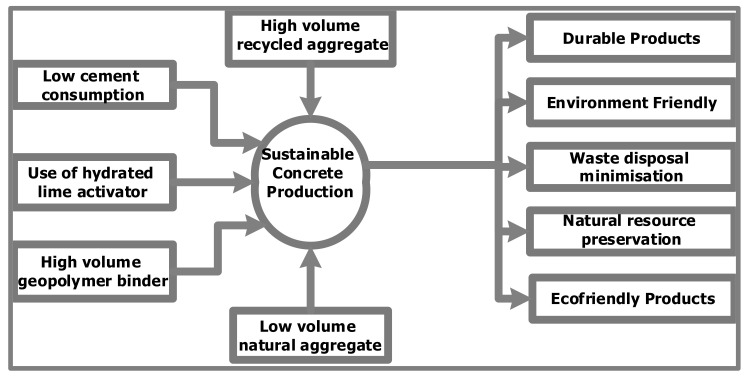
Sustainable concrete production approaches.

Figure 1 summarizes multiple strategies for creating more sustainable concrete alternatives that have been proposed by scholars [7,14,15] to try and reduce the unsustainability of concrete.

Numerous researchers have become interested in cutting-edge geopolymer technology and geopolymer composite production to attain sustainability in the manufacture of concrete. This is because geopolymers may be produced at low temperatures, using little energy, and by using a variety of wastes as either cementitious materials (SCM) or precursors [12]. Numerous scientists are fascinated by geopolymer concrete because it has the potential to be a more environmentally friendly alternative to ordinary Portland cement concrete [16,17,18]. It completely substitutes ordinary Portland cement with pozzolanic material (aluminosilicate-rich components).

Materials with silica and alumina-carrying phases have proven to be ideal for geopolymer synthesis [19]. To reduce the consumption of and dependence on cement, pozzolanic materials have become a major research focus in the field of cement and materials research in recent decades [20]. The most popular geopolymer precursors (aluminosilicate sources) that have been extensively studied thus far include fly ash, ground-granulated blast furnace slag, metakaolin, silica fume, and rice husk ash [21,22]. While many industrial by-products, agricultural wastes, and other waste products have been used as sources of aluminosilicate minerals, there has been comparatively little research on the use of either natural or spent diatomaceous earth as a geopolymer precursor. Its potential use as a precursor material in the creation of geopolymer concrete is made possible by its wide availability and pozzolanic properties.

Diatomaceous earth (DE), also known as diatomite or Kieselguhr, is derived from deposits made by the deposition of fossilized diatom skeletons, which are siliceous skeletons that are linked to clay minerals and quartz [23]. It is a lightweight mineral with a density of 0.25–0.50 ton/m^3^ possessing a high silica content (60–97%) and an amorphous porous structure [24]. Diatomaceous earth and other minerals containing amorphous SiO_2_ are practical and appealing materials for the creation of porous geopolymer materials and thus offer the construction industry a sustainable future. [25,26,27,28,29].

Diatomaceous earth has been used as a filtering agent, functional fillers in a variety of paints and plastics, in soil amendment, pesticides, in separation techniques, in nanotechnology, in capacitors, as a super hydrophobic substance, in pharmaceuticals, in biomedical applications, and as pore-forming agents in building materials [23,30]. Industries such as that of food processing and breweries generate a great deal of spent diatomaceous earth (SDE) as industrial waste [23,31,32]. For instance, the brewing industry generates approximately 378.1 million kilograms of SDE annually [33,34]. This spent diatomite is dumped in landfills or used as organic fertilizer in agriculture, which not only wastes land resources but also pollutes the environment [32,35]. Furthermore, the risk of leaching nitrogenous compounds present in the wasted diatomaceous earth could be increased by its use in agriculture. The regeneration of SDE might not be a practicable choice due to the high energy, labor, and financial requirements. Therefore, there is a significant interest in adopting SDE for other cost-effective and environmentally benign applications.

This review paper comprehensively presents the available literature on the incorporation of diatomaceous earth as a geopolymer concrete resource. A deeper understanding of its physical, chemical, and mechanical properties is accordingly required because—as a pozzolanic material—it is readily available in nature. Aside from that, it is also important to consider the performance characteristics of the concrete containing diatomaceous earth and its inclusion status in the literature. The purpose of this article is to bring together and disseminate scientific and technological knowledge to close the knowledge gap regarding the use of diatomaceous earth as a concrete raw material for sustainable development in the building sector.

## 2. Sustainability of Diatomaceous Earth as a Concrete Production Resource

Diatomaceous earth, being a natural pozzolanic substance, can be used as a supplementary cementitious ingredient. The use of active silica-rich materials, as supplemental cementitious materials (SCMs), has proved to be a viable alternative to Portland cement [36]. According to Snellings et al. [37], there are three advantages of using supplemental cementitious materials in the building and construction industries: the economic savings obtained by replacing cement with inexpensive natural pozzolans or industrial by-products, the diminished environmental effect related to greenhouse gas emissions generated during cement manufacture, and the improved end-product sustainability.

Diatomaceous earth’s carbon footprint with respect to its use as a bio-agricultural input was evaluated by [38,39]. It was found that the chemical fertilizer doses were reduced and thus became an alternative environmental management tool to contribute to the reduction in chemicals in the air, water, and soil. It was determined by Abrão et al. [40] that the amount of CO_2_ emitted was significantly reduced when Portland pozzolan cement blended with diatomaceous earth was used in concrete production.

Davidovits [41] revealed that geopolymer cement is more sustainable compared to Portland cement because it does not require high-temperature kilns, huge fuel expenditures, or large capital investments in plants and equipment during their manufacture.

This section examines the various ways that diatomaceous earth has been applied to advance concrete’s overall sustainability.

### 2.1. Diatomaceous Earth as a Cement Replacement Material

Li et al. [42] investigated the replacement of Portland cement (PC) in mortar and concrete mixtures with up to 40% highly reactive pozzolanic diatomaceous earth (DE) and found that a 30% by weight replacement of PC with DE was the optimum alternative, increasing strength development while reducing energy use and global warming potential by over 30%. Degirmenci and Yilmaz [43] demonstrated that the mortar’s compressive strength and sulfate resistance greatly increased while water absorption and mortar weight decreased when diatomite was used up to 15% by weight.

Diatomite powder, according to Ahmadi et al. [44], can replace up to 40% of the cement in mortars without compromising compressive strength while also enhancing tensile strength and transport characteristics. However, the mortars that had been amended with 15% calcined diatomite powder showed the best mechanical characteristics both at low and high temperatures [45]. The optimal percentage of diatomite replacement for cement, according to Macedo et al. [46], was determined to be 10%, since better results were yielded indicating that diatomite has a good potential as a partial substitute for cement in concrete construction. 

Regarding the use of waste marble powder (WMP) as an aggregate and up to 20% diatomite and fly ash as binders to partially replace natural hydraulic lime in mortars, Xu et al. [47] found that a 20% diatomite/fly ash addition improved mechanical properties, which was attributed to the pozzolanic reaction between the mineral admixtures and calcium hydroxide (Ca (OH)_2_) that mainly occurs during the curing period. Contrarily, Ergün [48] substituted diatomite and waste marble powder for cement, and the concrete containing 10% uncalcined diatomite and 5% waste marble dust was shown to have the best mechanical properties among its series. 

In addition to replacing cement, diatomite has also been used as a filler to act as a pore-forming agent in asphalt mixtures [49], magnesium phosphate cement [50], and straw fiber cement-based composites [51], where it improves the resistance to stripping, resistance to moisture damage, setting time, porosity, and thermal insulation, as well as facilitating the hydrogenation reaction.

Although the majority of studies came to the conclusion that using diatomite as a cement replacement material enhanced the mechanical properties, Pokorný et al.’s [36] investigation led to increased flexural strength and a decline in compressive strength. In contrast to a mechanical property analysis, Hasanzadeh and Sun [52] investigated the impact of cement replacement levels up to 10% on the transport properties and found that adding DE to cement paste increased viscosity while decreasing flow diameters, bleeding rate, setting times, and the heat of hydration.

### 2.2. Diatomaceous Earth as Lightweight Aggregate (LWA) Resource

Lightweight aggregates have a granular and porous structure, with a loose bulk density of less than 1.20 g/cm^3^ [53]. The use of lightweight aggregates with strong thermal insulation characteristics due to their porous structure can be a solution to improve the insulation potential of concrete elements, allowing for the avoidance of heavy building materials while adhering to thermal regulation standards [54]. According to [28,55,56], diatomaceous earth may be used as lightweight aggregates in mortar and concrete for insulating purposes because of its unique properties, such as its low density and porous structure, which are desirable for thermal performance, fire resistance, and sound absorption.

The strength and weight of the laboratory aggregates produced by Fragoulis et al. [57] after combining diatomite with 2–5% sawdust and pelletizing them at 1100 °C were comparable to commercial lightweight aggregates (LWAs). Additionally, the mechanical, physical, and thermal research findings by Posi et al. [58] determined that diatomite is a suitable lightweight aggregate for the production of pressed lightweight concrete blocks. Furthermore, Taoukil et al. [54] and Hasan et al. [59] investigated the feasibility of replacing sand in mortars with up to 100% diatomite; the results demonstrated that the thermal insulation capacity improved while the compressive and flexural strengths decreased.

The use of up to 40% of cement by volume as a binder in the manufacture of diatomite-based lightweight building elements was examined by Mehmedi Vehbi GÖKÇ [60], who found it to be inconvenient and recommended further research to bond diatomite more sustainably. However, according to Ünal et al. [61], lightweight concretes with diatomite can be utilized in buildings to achieve excellent insulation while reducing the structure’s self-weight or dead load. By combining diatomite with other aggregates and paraffin to create stable phase transition materials (PCMs), Xu and Li [62], Benayache et al. [63], and Costa et al. [64] also showed that the produced composites (PCMs) are promising candidates for thermal energy storage in buildings with maximum service temperatures of around 40 °C due to their high thermal resilience and energy storage capability.

Diatomite’s ability as a pore-forming agent for building elements such as waterproofing barriers [65], pyrophyllite support layers [66], and humidity control materials [67] has been investigated, and the results have demonstrated its excellent performance towards construction applications at low costs. Galán-Arboledas et al. [32] also attempted to replace clay, which is usually used to make bricks, with diatomaceous earth (DE) residues up to 10% by weight and noticed that doing so improved open porosity, lowered bulk density by up to 10%, and significantly reduced the flexural modulus to about 10 Mpa.

A mixture of low-grade diatomite (LDE) and oyster shells (OS) [68]; diatomite, rice husk ash, and sawdust [24]; diatomaceous earth and Brazil nut shells [69]; clay with kieselguhr [31,70,71]; and diatomite, sugar-filtered mud, and dolomite [72] were combined to produce porous refractory composites. The refractory products exhibited technical qualities that satisfied the expectations of porous and insulating materials, despite reductions in bending and compressive strength and the use of an unsustainable sintering technique.

### 2.3. Diatomaceous Earth’s Geopolymerization as a Source of Clinker-Free (Cementless) Concrete

Geopolymers are binding substances that differ from ordinary Portland cement (OPC) and are generated by activating silica and alumina-containing source materials (pozzolanic materials) with alkali solutions. This results in sodium aluminosilicate hydrate (N-A-S-H) gel or calcium aluminosilicate hydrate (C-A-S-H) gel products, which trigger the geopolymers’ hardening mechanism and produce materials with exceptional structural integrity and durability with the added benefit of lower greenhouse emissions [73,74]. By creating geopolymer construction composites with a lower carbon footprint, it is hoped that the growing concerns about global warming caused by emissions of carbon dioxide (CO_2_), a key greenhouse gas, from the ordinary Portland cement industry can be lessened [12,14]. 

**Figure 2:**
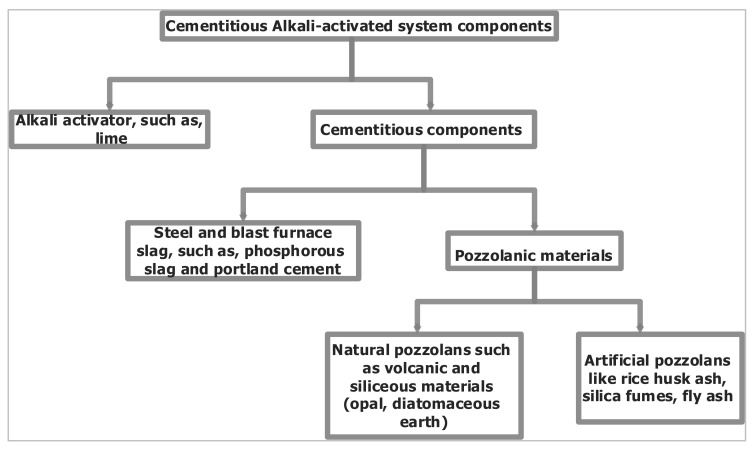
Geopolymer system’s components.

Figure 2 presents the geopolymer system’s components as summarized by Payá et al. [75].

**Figure 3:**
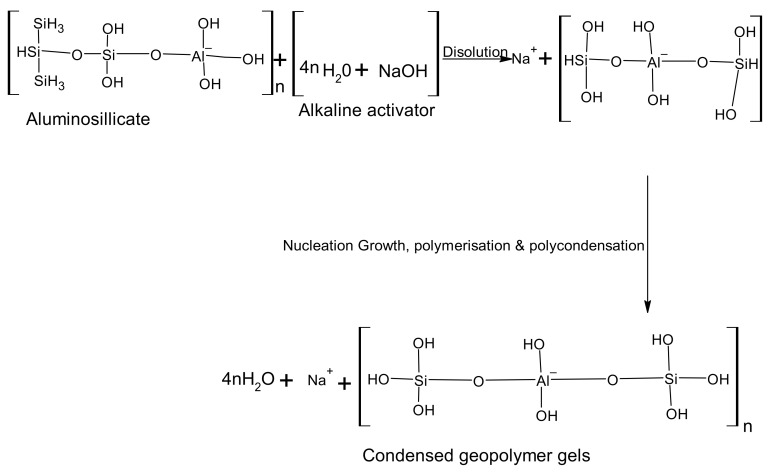
Geopolymerization process.

The fundamental processes of geopolymerization, presented in Figure 3, are the dissolution of solid aluminosilicate oxide in an M–OH solution, where M is an alkali metal (mostly Na and K); the dissolution of Al and Si complexes in an interparticle space; the formation of a gel phase by polymerization between silicate solution and Al and Si complexes; and the hardening of the formed gel phase at the end [73,76].

#### 2.3.1. Lime (Earth Alkaline) Activation of Diatomaceous Earth

Many ancient civilizations employed lime pozzolan concrete, and it is increasingly regaining prominence as an environmentally friendly alternative to cement for masonry and concrete applications [77] because of its abundance, low-cost production process, and ease of application [78]. Studies have demonstrated that lime activation is a pozzolanic reaction in which pozzolanic minerals react with lime in the presence of water to form cementitious compounds [79,80]. Quicklime (CaO) or hydrated lime (Ca (OH)_2_) can both be used to produce the desired result; therefore, naturally occurring pozzolana minerals containing silica and alumina have a high potential for lime activation. 

It was established through research on the production of concrete made of diatomaceous earth, lime, and gypsum[81] as well as diatomite and lime/limestone [27,82,83,84], that diatomaceous earth possesses a pozzolanic property that establishes it as a potential sustainable building material.

#### 2.3.2. Chemical Solutions as Diatomaceous Earth Activators

The interaction of solid aluminosilicates with a highly concentrated aqueous alkali hydroxide or silicate solution yields a synthetic alkali aluminosilicate substance known as a geopolymer, also known as an inorganic polymer or alkali-activated binder, with unique properties and characteristics, including a high compressive strength, high-temperature stability, and low thermal conductivity [73,76]. Sodium and potassium-based alkali activators are the most commonly used alkali activators, that is, a combination of sodium silicate (Na_2_SiO_3_) or potassium silicate (K_2_SiO_3_) and sodium hydroxide (NaOH)or potassium hydroxide (KOH) [85], although a combination of Na_2_SiO_3_ and NaOH have been widely used as activators in previous studies [76,86]. 

An alkali treatment of diatomaceous earth powder with the solutions of sodium hydroxide (NaOH) [87], potassium hydroxide (KOH) [88], and potassium silicate (K_2_SO_4_) [89] used to produce porous diatomite-based composites at ambient temperatures confirmed that diatomaceous earth can be exploited successfully as a silica source in geopolymeric systems. 

To produce porous silica ceramics, some researchers have attempted to activate diatomaceous earth using substances such as gelatin solution [90], boric acid [91], and polyethylene glycol (PEG) [92]. Although the products had good ceramic properties, the production methods are deemed unsustainable since they need high compaction pressures and sintering temperatures.

The effects of incorporating up to 40% calcined diatomite into high calcium fly ash geopolymer paste with sodium silicate (Na_2_SiO_3_) and sodium hydroxide (NaOH) solutions as alkaline activators were investigated by Phoo-ngernkhama et al. [93]. It was evident that the addition of diatomite accelerated the setting time, raised the strain capacity, and lowered the density of hardened paste; nonetheless, a diatomite substitution of 15% was determined to be optimal, yielding a compressive strength of 64.0 MPa.

### 2.4. Diatomaceous Earth Incorporation with Recycled Materials

Recycling waste plastics and agricultural residues is an excellent option for global sustainable development, and it is now a prevalent practice in the manufacturing sector [94,95]. Studies have demonstrated that various industrial and domestic wastes can be recycled and utilized as concrete elements in the development of green concrete [9].

Despite possessing good thermal and durability characteristics, geopolymers are brittle by nature and have a low resistance to tensile and flexural loadings, rendering them unsuitable for a range of structural applications [96]. Focusing on strengthening geopolymers with synthetic and natural fibers to boost their ductility and resistance to tensile stresses would help to solve this problem and enable the attainment of sustainability objectives.

#### 2.4.1. Polymeric Additives

Polymeric additives in the building sector may help to reduce raw material and energy consumption, as well as their environmental effects, while also assisting in the development of low-cost bricks with improved thermophysical qualities [97,98]. Regarding geopolymers, the addition of fibers can greatly enhance their mechanical and thermal characteristics [99].

Polyethylene, which is probably the most common type of plastic in the world, is the most commonly used synthetic material in the manufacturing of compressed earth blocks and other construction materials such as concrete [100,101,102]. 

The combined effects of waste Polyethylene Terephthalate (PET) particles and pozzolanic materials on the rheological, mechanical, and durability-realted properties of self-compacting concrete (SCC) revealed that waste Polyethylene Terephthalate (PET) particles can be reused as aggregates in concrete, although they decrease compressive, tensile, and flexural strengths while reducing the brittleness of concrete and the dead load of buildings due to their low unit weight [103]. The use of ultra-fine palm oil fuel ash (UPOFA) with shredded Polyethylene Terephthalate (PET) in concrete, according to Alani et al. [100], showed superior enhancement in terms, of porosity, initial surface absorption, gas permeability, water permeability, and rapid chloride permeability.

The change in the mechanical properties of concrete with the addition of high-density polyethylene (HDPE) plastics and fly ash in concrete was investigated by Venkateswara Rao and Srinivasa Rao [104]. It was observed that there was a drop in strength with the increasing HDPE content; however, the compressive strength of all the mixes fell within the acceptable strength range for most structural applications since the observed compressive strength was more than 20 MPa at 28 days. Nematollahi et al. [105] investigated the polyethylene (PE) and polyvinyl alcohol (PVA) fiber reinforcement of one-part geopolymers made using fly ash and blast furnace slag or lime, showing that geopolymer composite matrixes may be successfully created using polymeric additives of about 2%.

The majority of the existing research on the combined influence of plastic aggregates and pozzolanic materials on concrete characteristics has focused on the usage of polyethylene terephthalate (PET), whereas articles on high-density polyethylene (HDPE) and other forms of plastics are scarce. However, the practicality of stabilizing geopolymer binders (pozzolanic material), particularly those based on diatomaceous earth, with plastic wastes for the creation of sustainable construction materials has not been thoroughly studied, if at all.

#### 2.4.2. Natural Fibers

As a more sustainable alternative, natural fibers have been used in adobe and other traditional forms of earthen construction to reduce plastic shrinkage cracking and improve ductility, durability, and tensile and shearing strengths [106,107,108]. Generally, the use of natural fibers enhances the properties of structural building materials. Animal fibers are less desirable than plant fibers because large-scale animal fiber collection is more challenging [109] and, hence, not viable for large-scale production.

Plant fibers have been regarded to be excellent reinforcing elements for geopolymer matrices because geopolymerization occurs in high alkaline settings and lignocellulose fibers have a considerable tolerance to these conditions [110,111]; the addition of pozzolanic materials and alkaline activators in the composition of the geopolymer matrix act as specific fiber treatments [111]. Recent advances in the production of natural fiber-reinforced geopolymers as promising sustainable construction materials were reviewed by Silva et al. [110] and Li et al. [112]; a survey of successful reinforcements with natural fibers was reported, with a majority of the studies focusing on industrial by-products such as fly ash, ground-granulated blast furnace slag, construction and demolition wastes, and mine-tailings. Fibers act as concrete’s microcracking control units such that when the first fracture appears, the fibers function as bridges in the cracked portion, transmitting loads from the crack to the concrete, thereby making fiber-reinforced concrete more efficient [96,112,113]. 

Numerous investigations on geopolymeric composites reinforced with vegetable fibers have been published in the literature, including on cotton [114,115,116], bamboo [117], flax [118,119], sisal [111,116,120,121,122,123], coconut [120], and jute [110]. However, the interaction of vegetable fibers with a diatomaceous earth-based geopolymeric matrix has rarely been investigated.

In a literature review on the long-term mechanical properties of cellulose fiber-reinforced cement mortars that incorporated diatomite as a substitute for quartz sand, Ince et al. [124] discovered that pozzolans can enhance the durability properties of cellulose cement composites. 

## 3. Characterization of Diatomaceous Earth

Diatomaceous earth, which is a plentiful resource in many parts of the world, is a white to grayish rock that is porous, soft, and weakly bonded with a high SiO_2_ content, in addition to being amorphous with crystalline phases, (quartz, muscovite, and cristobalite), as well as possessing a high permeability of (0.1–10 mD) and a porosity of (35–65%) [56,125]. According to Mehmedi et al. [60] and other related researchers, natural diatomite is chemically composed of 67.80 to 90.07% silica (SiO_2_), 0.62 to 10.30% alumina (Al_2_O_3_), 0.20 to 6.85% iron oxide (Fe_2_O_3_), 0.05 to 1.21% titanium oxide (TiO_2_), 0.04 to 0.21% phosphate (P_2_O_3_), 0.19 to 3.0% limestone (CaO), 0.11 to 1.64% magnesium (MgO), 0.13 to 0.97% sodium (Na_2_O), and 0.13 to 1.47% potassium (K_2_O), wherein the first values are the minimum while the second values are the highest. Hasan et al. [126] defined diatomite as microparticles that can be utilized to substitute cement in concrete production, even though its silica content and structure differ greatly from one source to the next. 

Diatomite is made up of cylindrical particles with a square cell structure and a surface covered with micropores, which explains its high porosity and low density [46]. It has unique engineering properties including a high specific surface area, low dry density, high friction angle, high compressibility, and an unstable response under dynamic loads [127]. The high amount of amorphous SiO_2_ and accompanying pozzolana activity in diatomaceous earth is a significant aspect of its application in construction [27,84].

Diatomaceous earth, as determined by Reka et al. [125], represents a sedimentary rock of a biogenic origin; it is a soft solid, which can be easily disintegrated, with a white to greyish color, a bulk density of 0.51–0.55 g/cm^3^, a total porosity of 61–63%, and a specific gravity of 2.25 g/cm^3^. Diatomite’s industrial value is derived from its lightweight, low density, high porosity, high surface area, inertness, and high absorption capacity [24,128].

Modern analytical methods, such as the Nitrogen Adsorption Isotherm, X-Ray diffraction (XRD), Scanning Electron Microscopy (SEM), Transmission Electron Microscopy (TEM), Zeta potential, Thermal Gravimetric Analysis (TGA), and atomic absorption spectrophotometry [129]; Fourier-Transform Infra-Red (FT-IR) [63]; X-Ray fluorescence (XRF) [47,68,126]; wet chemical analysis (WCA) [91]; and inductively coupled plasma sector field mass spectrometry (ICP-MS) [69], which can be used to identify and characterize clay minerals, have been applied to diatomaceous earth by different researchers. 

It is evident from the sampled studies presented in Table 1 that the main chemical component of diatomaceous earth is silica (SiO_2_), which ranges between 56–93.5% wt, followed by alumina (Al_2_O_3_) between 0.05–12.28% wt, iron oxide (Fe_2_O_3_) between 0.23–26.4% wt, and calcium oxide CaO between 0.2–16.25% wt. Its other minor constituents are MgO (0.05–2.25% wt), MnO (0.005–0.22% wt), TiO_2_ (0.031–0.56% wt), K_2_O (0.09–2.28% wt), P_2_0_5_ (0.03–1.53% wt), and Na_2_O (0.1–5.69% wt). The loss on ignition (LOI) ranges between 0.35–14.65% wt. The review supports the claim made by Hasan et al. [126] wherein diatomite varies by geographic location.

According to Luhar [12], a pozzolanic material with properties such as a low calcium content, a high vitreous phase, possessing between 80 and 90% particle sizes that are less than 45 µm, a content of unburned material at less than 5%, a reactive silica content more than 40%, and less than 10% Fe_2_O_3_ content results in the optimal binding characteristics. Low-calcium binders are preferable for making geopolymers because the high amount of calcium can hinder the polymerization-setting rate resulting from an alteration of the microstructure [131]. Nyale [132] clarifies that a geopolymer binder is considered siliceous when the three key constituents, SiO_2_, Al_2_O_3_, and Fe_2_O_3_, total up to 70% or when their total and the reactive calcium oxide is less than 10%.

ASTM C618 [133] classifies a pozzolanic material that has a total content of SiO_2_, Fe_2_O_3_, and Al_2_O_3_ beyond 70% by weight and less than 10% CaO content by weight as a Class F normal type of pozzolan or aa silicate glass material.

According to the sampled data in Table 2, the earth-based concrete raw materials’ physical features are more of a concern than their mechanical properties. The bulk density (g/cm^3^) ranges between 0.32–0.767, the porosity (%) is between 73–77, and the specific gravity is about 1.9.

To produce geopolymer concretes, scientists have used a variety of precursors, including fly ash (FA), rice husk ash (RHA), ground-granulated blast furnace slag (GGBS), silica fume (SF), and palm oil fuel ash (POFA). Table 3 displays the properties and compositions of these precursors.

When diatomaceous earth is compared to the materials shown in Table 3, it seems to be the lightest, with a similar chemical composition to Rice husk ash (RHA), Silica fume (SF), and Palm oil fuel ash (POFA).

## 4. Production Process of Diatomaceous Earth-Based Concrete

The fundamental components used in the manufacture of geopolymer elements are pozzolanic material and activator additives used to accelerate the hydration process [73]. In the literature under review, some researchers used either the one-part or the two-part geopolymer preparation approaches. 

For the one-part approach, all of the ingredients (the precursor material and the solid activator) are dried and mixed uniformly, after which water is gradually added to the mixture while stirring slowly [134,135,136]. 

The manufacturing parameters for lightweight concrete discussed in Section 2.2 are displayed in Table 4. 

The research by [24,32,57,69,75] seems to be unsustainable given the high compaction pressure and high sintering temperatures needed by their concrete production systems. Cost sustainability may be a challenge for Reka et al. [27], Galán-Arboledas et al. [32], and Pimraksa and Chindaprasirt [81], due to the need for extrusion and autoclaving equipment.

Even though Loganina et al.’s [83] experiment was carried out at room temperature without the use of compaction pressure, the output specimens were feeble, reaching a maximum strength of 3.92 MPa after a 28-day curing period. To bind the lightweight diatomaceous earth aggregates together, [54,60,61] employed cement as a binder. In addition to using cement as a binder, Hasan et al. [59] had to pelletize diatomaceous earth at a temperature of 650 °C. 

In the case of a two-part geopolymer preparation, the activator solution is prepared 24 h beforehand, added to the dry precursor material, and mixed until homogeneity is obtained. Subsequently, molding and curing follow [137]. The production parameters for the two-part geopolymer mixes discussed in Section 2.3 are shown in Table 5. 

The most often utilized alkali activators are those based on sodium and potassium. Previous research has demonstrated that sodium-based alkali activators have a higher activation efficiency than potassium-based activators [86].

Using potassium hydroxide (KOH) as an alkaline activator and diatomite as a precursor, Nakashima et al. [88] and Bagci et al. [89] developed promising geopolymer specimens with a maximum strength of 5.78 MPa and 71 MPa, respectively. The sustainability potential of the obtained geopolymers was diminished by the high compaction pressure and high-temperature sintering methods used by Matsunaga et al. [90], Šaponjić et al. [91], and Akhtar et al. [92]. 

The alkaline activation of metakaolin with sodium silicate solution plus NaOH was reported by Elahi et al. [22] to produce better compressive strength than in the samples activated with NaOH alone. Fernandez and Palomo [138] reported more than twice as much strength for FA-based concrete when activated by NaOH and water glass in combination instead of by NaOH alone. Sodium silicate has been known to act as an activator that enhances the polymerization process resulting in a silica-rich reaction product and, hence, improving the strength of geopolymers.

Researchers have demonstrated the effectiveness of lithium hydroxide solution as an alkali initiator; lithium can be coated with geopolymer particles to reduce the dissolution of active silica, and the chance that dissolved active silica will form an Alkali–silica reaction (ASR) gel. With the use of solid Na_2_CO_3_ and hydrated lime as activators of fly ash and silica fume-based geopolymers, the strength of about 50 and 85 MP was obtained in 28 d at curing temperatures of 25 and 85 °C, respectively [86].

The ratio of Na_2_SiO_3_ to NaOH (SS/SH) is another effective factor that has been found to govern the compressive strength (CS) of geopolymer concretes (GPC). The effective ratio of SS/SH used to prepare GPC with a sufficient CS fall in the range of 1.0 to 3.0, with 2.0 being the most frequently and effectively employed [139]. The best NaOH solution molarity value for the alkali solution was found to be 12 in other studies while some studies determined 14 as the optimum molarity [140].

Although high-performance geopolymers were developed by Phoo-ngernkhama et al. [93], it is difficult to determine the potential of diatomite in the mixture because it only used up to 40% of high calcium fly ash. The same goes for Font et al. [87], who substituted diatomite for rice husk ash. It has also been noted that the water to geopolymer solids ratio by mass is very important in the design of a geopolymer concrete mix [141]

**Figure 4:**
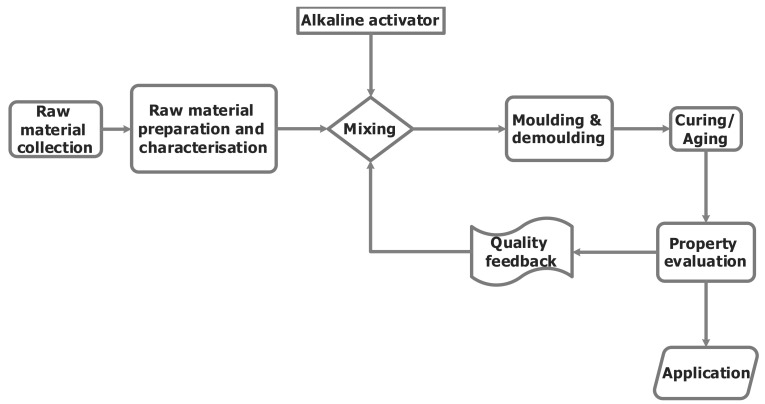
General geopolymer production process adopted by most of the researchers.

Figure 4 depicts the general geopolymer production system adopted by most of the researchers.

The curing process is one of the most important phases in the synthesis of geopolymers since it has a substantial impact on the final product’s features. Depending on the system of alkali-activated materials, heating (thermal curing or oven), sealing (wrapping), steaming, and water immersion are the usual techniques used to achieve optimum properties [21]. Researchers have tried several curing techniques for geopolymer concrete, including heating in an oven, membrane curing, steam curing, hot gunny curing, hydrothermal curing, room temperature curing, and water curing; of these, oven curing turned out to be the most effective [140]. Thermal curing within the first 3 days has been widely advocated to increase the chemical reactivity at the first hardening stages, preferably at temperature ranges of 60–80 °C [142], 60 to 90 °C [143], 80 to 90 °C [138], 40 to 85 °C [144], 40 to 100 °C [145], and 40 to 90 °C [146] during the initial 24 h. Thermal curing has been shown to minimize porosity and provide a considerable strength gain, according to recent research [147,148,149]. Optimal curing temperatures, such as 50 [89], 60 [17,150,151], 70 [88,152], and 80 °C [153] have been proposed by several studies.

Practical applications, according to Abdullah, Ibrahim [152], do not require heat curing to last more than 24 h since the rate of strength rise is rapid up until a certain point; however, beyond 24 h, the rate of strength increase is only modest. After a 24-hour thermal curing, and to avoid an unfavorably high degree of water evaporation during the setting of the geopolymer binder, which decreases the strength and causes sample breaking, geopolymer products are cured under controlled humidity at an ambient temperature [99,154,155,156].

Table 6 presents the basic standards and procedures that were employed by the researchers in the process of developing the different concrete mixes.

## 5. Performance Properties of Mixtures Incorporating Diatomaceous Earth

A substantial body of research has demonstrated that the properties of concrete from alkaline-activated binders depend on a wide range of variables, such as the physical and chemical composition of the source materials, the type and concentration of the alkaline solution, the mixing ratios, and curing regimes. As a result, the vast majority of research has largely focused on the microstructural and mechanical properties of hardened concrete as well as the durability aspects [22,167]. 

For structural work and safety assessments, mechanical characterization, which includes compression strength, flexural strength, shear strength, and hardness testing, is required. In particular, compressive strength and durability tests are regarded as key indicators of the viability of masonry [168,169]. The physical attributes are also more important since they can be used to predict shrinkage, apparent bulk density, size or texture, moisture content, porosity, permeability, adhesion, and thermal properties [170]. 

Table 7a,b illustrate categorically that the most frequent characteristic properties taken into account in the majority of the studies under review are porosity, bulk density, thermal conductivity, and compressive strength. The concrete mixes containing diatomaceous earth appear to be porous, with porosity varying from 25 to 92.5%; lightweight, with densities falling between 0.37 and 1.81 g/cm3, with low thermal conductivity ranging from 0.09 to 0.45 W/Mk; and the majority of them exhibit noticeably high compressive strengths. Additionally, it can be seen that using diatomite as an alkaline activated binder rather than a lightweight aggregate can result in stronger concretes.

According to Cong and Cheng [86], the durability of geopolymers is not only affected by their strength but also their resistance to harsh environments, such as their abrasion performance, porosity, chemical erosion resistance, dry shrinkage, carbonization resistance, and other parameters.

Verma et al. [13] report that geopolymer concrete has better physical, mechanical, and durability properties than Portland cement concrete and it is highly resistant to acid, sulphate, and salt attacks. The performance properties of concrete reported by various studies in Table 7a,b agree with the findings of Luhar [12] that geopolymer’s properties may differ not only by the origin, morphology, and particle size of the binder but also by the metal, alkali, and amorphous contents. It has been established that the properties of geopolymers also depend on various parameters such as the alkaline activator concentration, alkaline solution to binder ratio, sodium silicate to sodium hydroxide ratio, alkaline liquid to binder ratio, curing duration, curing temperature, superplasticizer dose, water to binder ratio, and the curing period.

The required 28-day compressive strength values for normal and heavyweight concrete, as specified by the European Standard EN 206-1 [171] are 8–100 and 10–115 MPa for cylindrical samples with a diameter of 150 mm and a height of 300 mm and a cubic sample with a side length of 150 mm, respectively. The specifications of 8–80 and 9–88 MPa are similar requirements for lightweight concrete [136]. According to Mackenzie and Welter [99], the compressive strengths of geopolymer matrix materials span a wide range, from 1 MPa for the very weak products of solid-state synthesis through 26 MPa for sol-gel synthesized geopolymers to 110 MPa for a product synthesized from fly ash activated with sodium silicate and NaOH solution. 

Table 8 shows the standard test procedures that were referenced for the evaluation of the various performance properties. 

## 6. Conclusions

This literature review demonstrates the use of diatomaceous earth as one of the efficient methods for developing lightweight, insulating, and sustainable building materials while reducing the negative economic and environmental effects of industrial solid wastes; nevertheless, further research is needed to sustainably use diatomaceous earth and other additives in the construction sector. This comprehensive survey of the information from the literature on the issue of diatomaceous earth’s incorporation in geopolymer concrete development is concluded as follows:

❖ Diatomaceous earth is one of the naturally occurring pozzolanic materials, which finds applications in the development of geopolymers, as shown in Figure 2; however, there has been relatively little research to determine its viability as a geopolymer binder to create cementless (clinker-free) concrete. Most of the studies have concentrated on its integration either as a partial cement replacement or replacement for fine aggregate.
❖ Although silica-based raw materials have been the subject of many studies as resources for geopolymeric concrete, the reinforcement of geopolymeric concretes, particularly those based on diatomaceous earth, with either natural or synthetic fibers or a combination of the two, has not been addressed.
❖ Despite the authors’ keen interest in the mechanical, physical, and thermal qualities of building materials, this review showed that there has been remarkably little effort made towards theoretical property correlation modeling for performance prediction. Accordingly, the review undertaken by Mohammed et al., [140] revealed the same concern.

## Figures and Tables

**Table 1:** Chemical compositions for several reported diatomite analyses.

S/N	Major Oxides (% wt.)											Reference
	SiO_2_	Al_2_O_3_	Fe_2_O_3_	CaO	MgO	MnO	TiO_2_	K_2_O	P_2_O_5_	Na_2_O	LOI	
1.	86.3	2.9	1.7	0.8	0.2	-	0.5	0.5	0.5	0.3	6	[32]
2.	92	0.05	0.82	-	-	0.08	0.11	0.34	0.04	0.55	6.01	[44]
3.	82.02	3.76	5.14	2.61	0.6	-	0.35	0.2	0.73	0.33	2.35	[45]
4.	70.77	6.61	9.02	1.14	-	0.22	-	0.51	-	-	-	[46]
5.	71.35	4.87	7.98	11.71	-	-	-	3.1	-	-	-	[47]
6.	68.67	10	3.54	10.71	0.68	-	-	0.71	-	-	5.69	[48]
7.	83.48	11.51	1.82	0.163	0.554	0.01	0.353	1.81	0.04	-	-	[50]
8.	80.13	5.43	1.23	0.53	-	-	-	-	-	-	12.66	[51]
9.	93.5	1.6	1.1	0.4	0.05	-	-	0.09	-	2.51	0.35	[52]
10.	79.86	9.01	6.29	0.51	0.47	-	0.56	2.28	0.1	0.16	0.6	[58]
11.	67.2	10.09	2.74	1.36	0.63	-	-	0.67	-	0.36	10.3	[61]
12.	60.71	7.9	1.17	16.25	0.4	0.04	0.25	1.06	-	-	14.65	[63]
13.	71.16	12.25	2.16	0.39	0.5	-	-	0.76	-	0.34	10.3	[68]
14.	70.65	4.26	1.07	0.83	2.25	0.01	0.52	1.67	1.53	5.69	11.1	[69]
15.	73.68	12.28	3.29	0.7	0.44	-	-	1.01	-	0.12	8.26	[91]
16.	56	6.5	26.4	-	-	-	-	-	-	-	-	[126]
17.	89	0.64	0.23	0.2	0.07	0.005	0.031	0.11	0.03	0.1	9.1	[130]

**Table 2:** Mechanical and Physical properties of diatomaceous earth.

S/N	Bulk Density (g/cm^3^)	Porosity (%)	Specific Gravity	Water Absorption (%)	Dry Compressive Strength (MPa)	Reference
1.	0.32–0.64	-	-	-	-	[28]
2.	0.559	77	-	-	-	[54]
3.	0.6	-	1.85	-	-	[58]
4.	0.767	-	1.9	6.5	-	[126]
5.	0.55–0.60	73–75	-	-	3.4–4.6	[130]

**Table 3:** Characteristics of other geopolymer precursor materials.

Property	Fly Ash (FA)	Rice Husk Ash (RHA)	Ground-Granulated Blast Furnace Slag (GGBS)	Silica Fume (SF)	Palm Oil Fuel Ash (POFA)
Bulk Density (g/cm^3^)	1.3	0.96–1.6	1.2	1.35–1.51	2.4–2.5
Specific gravity	2.2	2.11	2.9	2.2	2.14
Silica (SO_2_)	38–55	>90	30–40	>85	>80
Alumina (Al_2_O_3_)	20–40	>9	5–20	<2	16–18
Iron oxide (Fe_2_O_3_)	6–16	>2.8	<2	<1	8–10
Calcium oxide (CaO)	1.8–10	1–2.2	35–40	-	5–18
Magnesium oxide (MgO)	1–5	>1	5–18	-	>1.2

**Table 4:** The manufacturing parameters for lightweight concrete with diatomite as a resource.

S/N	Water: Binder	Specimen Size (mm)	Compaction Pressure (MPa)	Curing/Sintering	Reference
1.	0.4	220 × 110 × 65	10	1200 °C	[24]
2.	0.4	Ø16–10	10	Autoclaved—130 °C—3 h	[27]
3.	-	120 × 28 × 18 Ø85 × 10	Extrusion	850–1050 °C	[32]
4.	0.5–0.7	250 × 250 × 20 160 × 40 × 40	-	Room temperature	[54]
5.	0.5	Ø5–10 Ø10–15 Ø15–20	-	1100 °C 12–15 min	[57]
6.	2	50 × 50 × 50	0.85	Room temperature	[58]
7.	0.55–1	Ø150 × 300 150 × 150 × 600	-	Room temperature	[59]
8.	2.175–7	100 × 100 × 100	-	Room temperature	[60]
9.	0.15	100 × 100 × 100		Room temperature	[61]
10.	2	60 × 8 × 6	15	700–900 °C	[68]
11.	-	Ø35 × 35	15	750–950 °C	[69]
12.	-	60 × 8 × 6	15	800 °C	[72]
13.	0.1	35 × 75 × 150	3.5	Autoclaved—0.14 Mpa 130 °C—4 h	[81]
14.	0.56	30 × 30 × 30	-	Room temperature	[83]

**Table 5:** Preparation of diatomite-based geopolymer mixes with diatomite as a precursor.

S/N	Alkaline Activator	Specimen Size (mm)	Compaction Pressure (MPa)	Curing	Reference
1.	NaOH	Mortar pastes	-	20 °C for 28 days	[87]
2.	3M KOH	40 × 40 × 13 Ø5 × 13	6.25	70 °C for 24 h	[88]
3.	KOH	10 × 10 × 100 Ø3 × 6	-	50 °C for 24 h	[89]
4.	Gelatine	Ø40 × 18	40	1150–1350 °C—2 h	[90]
5.	Boric acid	Pellets	40	1150 °C	[91]
6.	Polyethylene glycol (PEG)	Ø10 × 10	50 MPa	900–1400 °C	[92]
7.	Na_2_SiO_3_/ 10M.NaOH=2	50 × 50 × 50 Ø50 × 100	-	60 °C for 24 h	[93]

**Table 6:** Standard Procedures for raw material and concrete preparation.

S/N	Characterization Type	Standard
1.	Standard Specification for Raw or Calcined Natural Pozzolan for Use in Concrete	ASTM C618 [133]
2.	Particle size distribution	ASTM D6913 / D6913M-17 [157] ASTM C136/C136M – 14 [158]
3.	Chemical characterization	ASTM C114-10 [159]
4.	Pozzolanic nature of earth material	ASTM C311/C311M [160]
5.	Specific gravity	ASTM-D854 [161]
6.	Loss on ignition	ASTM D 7348-13 [162]
7.	Water to binder ratio for normal consistency	ASTM C187 [163]
8.	Mixture preparation of mortars and concrete	ASTM C305-14 [164]
9.	Determination of the initial and final setting times	ASTM C191-08 [165]
10.	Making and Curing Concrete Test Specimens	ASTM C31-19 [166]

**Table 7:** (**a**): Performance properties of concrete when diatomaceous earth is incorporated as a lightweight aggregate. (**b**): Performance properties of concrete when diatomaceous earth is incorporated as a clinker-free geopolymer resource.

(**a**)								
S/N	Porosity (%)	Density (g/cm^3^)	Thermal Conductivity W/Mk	Sound Transmission km/s	Compressive Strength MPa	Water Absorption %	Flexural Strength MPa	Reference
1.	74.28–92.45	0.37–0.6	0.0878–0.1035	15.78–17.35	-	-	-	[24]
2.	58.53	0.71–0.91	-	-	14.7–19.4	52.63	-	[27]
3.	30	1.64	0.45	-	16.3	-	6	[32]
4.	47.08	1.14	0.16	-	7.66	37.19	2.4–0.74	[54]
5.	-	0.55–0.79	-	-	-	-	-	[57]
6.	58–61	1–1.2	0.15–0.19	-	7.8–12.9	61–72	-	[58]
7.	-	1.81	-	-	28	21.1	-	[59]
8.	45	-	-	61.3	4.29	-	-	[60]
9.	-	0.9–1.19	-	-	2.5–8	-	-	[61]
10.	50.2	1.26	-	-	18.8	-	-	[68]
11.	49	1.06	0.2	-	8.5	9	-	[69]
12.	50.39	1.25	-	-	-	-	10.05	[72]
13.	-	0.73	0.13	-	17.5	46	-	[81]
14.	-	-	-	-	3.92	-	-	[83]
(**b**)								
**S/N**	**Porosity (%)**	**Density (g/cm^3^)**	**Thermal** **Conductivity W/Mk**	**Sound** **Transmission km/s**	**Compressive Strength MPa**	**Water** **Absorption %**	**Flexural Strength MPa**	**Reference**
1.	-	-	-	-	30	-	-	[87]
2.	-	-	0.171	-	5.78	-	-	[88]
3.	-	-	-	-	71	-	9.2	[89]
4.	-	-	0.09–0.16	-	-	-	-	[90]
5.	68	-	-	-	-	-	-	[91]
6.	25	1	-	-	106	-	-	[92]
7.	-	1.76	-	-	17.24	-	-	[93]

**Table 8:** Standard test methods referred to in the literature.

S/N	Property Tested		Standard Test Method
1.	Physical	Density Porosity Water absorption Linear Shrinkage	ASTM-C642 [172] ASTM C20-00 [173] ASTM C373-14 [174] ASTM C326-09 [175]
2.	Mechanical	Compressive strength Flexural strength	ASTM C109/C109M [176] ASTM C773-88 [177] ASTM C78/C78M – 02 [178]
3.	Insulation	Thermal conductivity	ASTM C 177 [179]
		Pulse velocity	ASTM C597 [180]

## Data Availability

The data presented in this study are openly available in the references that were cited accordingly.
